# Analyzing COVID-19 and Air Pollution Effects on Pediatric Asthma Emergency Room Visits in Taiwan

**DOI:** 10.3390/toxics12010079

**Published:** 2024-01-17

**Authors:** Yan-Lin Chen, Yen-Yue Lin, Pi-Wei Chin, Cheng-Chueh Chen, Chun-Gu Cheng, Chun-An Cheng

**Affiliations:** 1 School of Medicine, National Defense Medical Center, Taipei 11490, Taiwan; a0931994342@gmail.com; 2 Department of Emergency Medicine, Taoyuan Armed Forces General Hospital, Taoyuan 32549, Taiwan; 3 Department of Emergency Medicine, Tri-Service General Hospital, National Defense Medical Center, Taipei 11490, Taiwan; 4 Department of Nursing, Ministry of Health and Welfare, Hua-Lien Hospital, Hua-Lien 97061, Taiwan; pipi610715@gmail.com; 5 Department of General Surgery, China Medical University Beigang Hospital, Yunlin 65152, Taiwan; d2616@mail.bh.cmu.edu.tw; 6 Department of Neurology, Tri-Service General Hospital, National Defense Medical Center, Taipei 11490, Taiwan

**Keywords:** pediatric asthma emergency room visits, COVID-19 pandemic, air pollution

## Abstract

(1) Background: An asthma exacerbation that is not relieved with medication typically requires an emergency room visit (ERV). The coronavirus disease 2019 (COVID-19) pandemic began in Taiwan in January of 2020. The influence of the COVID-19 pandemic on pediatric ERVs in Taiwan was limited. Our aim was to survey pediatric asthma ERVs in the COVID-19 era; (2) Methods: Data were collected from the health quality database of the Taiwanese National Health Insurance Administration from 2019 to 2021. Air pollution and climatic factors in Taipei were used to evaluate these relationships. Changes in the rates of pediatric asthma ERVs were assessed using logistic regression analysis. Poisson regression was used to evaluate the impact of air pollution and climate change; (3) Results: The rate of pediatric asthma ERVs declined in different areas and at different hospital levels including medical centers, regional and local hospitals. Some air pollutants (particulate matter ≤ 2.5 µm, particulate matter ≤ 10 µm, nitrogen dioxide, and carbon monoxide) reduced during the COVID-19 lockdown. Ozone increased the relative risk (RR) of pediatric asthma ERVs during the COVID-19 period by 1.094 (95% CI: 1.095–1.12) per 1 ppb increase; (4) Conclusions: The rate of pediatric asthma ERVs declined during the COVID-19 pandemic and ozone has harmful effects. Based on these results, the government could reduce the number of pediatric asthma ERVs through healthcare programs, thereby promoting children’s health.

## 1. Introduction

### 1.1. Coronavirus Infection since 2019

New respiratory and related diseases in Wuhan City, Hubei Province, China, have been caused by coronavirus infection since December 2019. Most patients have clinical findings of fever, difficulty breathing, and infiltrative lesions in both lungs on chest radiography [1]. Coronavirus disease 2019 (COVID-19) was first identified in Taiwan on 21 January 2020. Patients who meet the clinical and laboratory criteria, and have moderate or severe complications, must be admitted and treated immediately [2].

### 1.2. Epidemiology of Asthma

Asthma is a chronic airway obstruction caused by many factors. Genetic factors combined with specific environmental factors may result in bronchial wall thickening and respiratory over-responsiveness. Air pollution, daily temperature changes, infections, emotional changes, strenuous exercise, drugs, preservatives, allergic agents, and other factors can cause rapid inflammation of the respiratory tract, induce bronchial constriction, and increased sputum formation [3].

Symptoms of asthma exacerbation include shortness of breath and/or chest tightness. Airflow obstruction increases without immediate treatment, causing increased breathing work and respiratory muscle fatigue, resulting in hypercapnic and hypoxemic respiratory failure due to gas exchange inefficiency. Male patients with asthma, the elderly, and children were less sensitive to the symptoms. As such, an acute asthma attack is a significant public health problem that affects patients and their parents or caregivers and is associated with decreased quality of life, decreased school attendance, emergency room visits (ERVs), and hospitalizations, as well as a higher mortality risk [4]. Based on information in the Taiwanese National Health Insurance database, the prevalence of asthma among adults has been gradually increasing from 2000 to 2011; the prevalence of asthma among adults increased from 7.57% in 2000 to 11.53% in 2011 [5]. However, the overall prevalence of asthma in Taiwan was 9% in 2017 and 2018 [6]. The prevalence of asthma is higher in children; however, the morbidity and mortality rates due to asthma are higher in adults. Childhood asthma is more common in boys than girls, and several environmental factors have been identified. In children, asthma may impair airway development and lead to reduced lung function as an adult [7]. The prevalence of asthma in the United States is 7.7%, with 4.6 million children under the age of 18 years [8], and up to 36% ERVs of asthmatic children under 18 years of age were affected by an asthma attack in the United States in 2016 [9].

Asthma affects approximately 262 million patients worldwide, thus causing about approximately 461,000 deaths in 2019 [8]. Acute asthma attacks are among the most common reasons for ERVs in all age groups and impose a significant burden on healthcare systems. A previous study showed that the proportion of children with asthma aged 6–12 years who were diagnosed by a doctor in Taiwan was 11% between 2008 and 2009, 10.9% between 2010 and 2012, and 9% between 2017 and 2018 [6]. Based on the self-reported questionnaire data from the National Health Interview Survey (NHIS) by the Ministry of Health and Welfare National Health Service in Taiwan in 2017, the overall prevalence of asthma was estimated to be 4.3% in all age groups, including children and adults, which translated into approximately 2 million patients and 318,000 pediatric patients [10]. A total of 9.2% of children < 12 years of age are sent to the emergency room for asthma exacerbations every year, and 23% of people have an asthma attack each year; however, 17% do not follow regular outpatient visits [10]. The five most common causes of asthma in children under 12 years of age are viral infections (56.7%), dust mites (44.5%), sudden temperature changes (42.7%), air pollution (24.6%), and colder food (21.6%) [10].

### 1.3. Air Pollution and Pediatric Asthma

The respiratory disorders were associated with particulate matter, nitrogen oxides, and sulfur dioxide (SO_2_) [11,12]. The asthma outcomes in childhood were related to particulate matter ≤ 2.5 µm (PM_2.5_), nitrogen dioxide (NO_2_), and ozone in the previous studies [13,14,15,16,17]. Numerous studies have evaluated the effects of PM_2.5_ and O_3_ on children with asthma in Taiwan and Hong Kong before 2019 [18,19,20,21]. PM_2.5_ induces more tissue kallikrein of epithelial cells with mucus hypersecretion and barrier function and causes immunoglobin E, eosinophils to increase [13,14]. Ozone is produced via a photochemical reaction between nitrogen oxides and volatile organic compounds in the troposphere, with motor vehicle emissions being the greatest source [22]. High ozone concentrations can irritate the eyes and nose and cause urgent respiratory distress with impaired lung function [23]. Daily changes in ozone concentrations are positively associated with the incidence of asthma and daily non-accidental mortality [24]. The effects of O_3_ on asthma risk in Taiwanese schoolchildren have been reported to have an adjusted OR of 1.14 [23]. Ozone is a strong oxidant that causes oxidative damage to the airway cells and induces inflammatory reactions in the airways. Acute ozone exposure in young male children resulted in an increase in sputum neutrophils in 30% of children [25]. Increased ozone induced type 2 inflammation [13]. Controlling ozone pollution is challenging in the United States in 2016, where 90% of the substandard national ambient air quality conditions were caused by ozone, whereas only 10% were caused by particulate matter and other regulated pollutants [24]. Globally, ozone causes premature deaths and several million asthma-related ERVs annually. To combat global ozone pollution, more aggressive reductions in fossil fuel consumption are needed to reduce NO, volatile organic compounds, and greenhouse gas emissions [24]. In Taoyuan, Taiwan, the RR of the ERVs for asthma after ozone exposure was 1.2 [26].

### 1.4. Other Infection Decline during COVID-19 Pandemic

Infections can induce acute bronchospasms and increase sensitization to environmental air pollutants, which are risk factors for asthma exacerbation in children [27]. A reduction in respiratory pathogens is considered the most important factor in reducing asthma exacerbation. The reasons for the reduction in adult asthma exacerbations are multifactorial, with fewer viral infections resulting from fewer social contacts and the use of monoclonal antibodies in the United States [28]. A decrease in retroviral infection rates has been observed in the United States, and the reduction in infections has the potential to decrease asthma exacerbations [29]. The total number of outpatients and ERVs for influenza-like illnesses in Taiwan is expected to significantly decrease by 2021 [30]. Pediatric ERVs for respiratory disease decreased during the COVID-19 pandemic in Kaohsiung [31]. It may have had fewer asthma exacerbations because of protective measures such as maintaining social distancing, wearing face masks, and frequent hand washing. Residents’ activities decreased after the COVID-19 outbreak. Decreased economic activity and air pollution have health benefits [32]. Lifestyle changes with school closures and stay-at-home orders, as well as restricting eating outside, are measures that reduce air pollution and person-to-person contact.

### 1.5. The Emergency Room Visits Decline during COVID-19 Pandemic

During the COVID-19 pandemic, the number of ERVs in Taiwan have decreased significantly [33,34]. In the early stages of the pandemic, the number of daily ERVs was significantly reduced and examination times were shortened. Hospitalized patients have benefited from the pandemic, with shorter emergency room stays, faster discharges, lower mortality rates, and fewer readmissions [34].

The government imposed restrictive measures on Taiwan. Pediatric asthma exacerbations decreased during the COVID-19 period compared to the previous 5 years in northern Italy [35]. There are fewer ERVs and hospitalizations of children with asthma in the United States and Canada [29,36]. The reasons for this reduction in asthma ERVs are multifactorial and may include decreased viral infections, restrictive measures, and reduced traffic-related air pollution (TRAP) [35,36]. However, there is a lack of information regarding pediatric asthma ERVs in Taiwan, and this issue requires further study.

This study aimed to determine the impact of the COVID-19 pandemic on ERVs in pediatric patients with asthma in Taiwan. We assessed whether the rate of pediatric asthma ERVs was reduced during the COVID-19 pandemic compared to that in the pre-COVID-19 period. In addition, we examined the influence of different air pollutants on pediatric ERVs for asthma during these two periods. By understanding the possible risk factors for pediatric asthma ERVs during the COVID-19 pandemic, governments can promote children’s health through aggressive policies in the post-COVID-19 era.

## 2. Materials and Methods

Data were collected from the Taiwanese National Health Insurance Administration and the National Health Insurance database. Pediatric asthma was defined as a patient < 18 years old patients who had an ICD10-CM of J45; had 4 outpatient visits within 1 year; used an asthma medication; and had an Anatomical Therapeutic Chemical classification code of R03AC02, R03AC03, R03AC04, R03AC06, R03AC12, R03AC13, R03AC16, R03AC18, R03BA01, R03BA02, R03BA05, R03BA08, R03AK06, R03AK07, H02AB06, H02AB07, R03DC01, R03DC03, or R03DA05; had an ERVs; or had a hospitalization. The rate of pediatric asthma ERVs was defined as the number of pediatric asthma ERVs divided by the number of pediatric asthma patients, of which it was published every season in the healthcare quality information dataset [37].

We collected data on the rates of pediatric asthma ERVs from the first season of 2019 to the fourth season of 2021. The first season was from January to March, the second season was from April to June, the third season was from July to September, and the fourth season was from October to December. The change in the rate of pediatric asthma ERVs before the COVID-19 pandemic (2019) and during the COVID-19 pandemic (2020–2021) was compared based on the regional distribution and hospital levels (including medical centers, regional and local hospitals). Since the trend of annual pediatric asthma ERVs in Taipei District (including Taipei City and New Taipei City) is relatively close to the trend of pediatric asthma ERVs in Taiwan, Taipei District was selected to study the risk of pediatric asthma ERVs due to air pollution and climate factors. Monthly air pollution data were obtained from the Taiwan Air Quality Monitoring Network of the Ministry of Environmental Protection [38]. The climate factors were collected from the Taipei City Statistical dataset [39]. We used monthly air pollution data from the Wanhua station, located in the Taipei Basin Center. Wanhua Station is located at Fuxing Elementary School (latitude and longitude: 25.04679 and 121.50888, respectively; Google Maps). The values of each air pollutant, including PM_2.5_, NO_2_, O_3_, and climate factor including ambient temperature and relative humidity, were analyzed for correlations. If the *p* value of the coefficient of the two parameters was < 0.05, indicating a higher correlation, one of the values needed to be excluded. To reduce the likelihood of a multiple collinearity problem, we screened variables by setting the inclusion criteria at |r| < 0.8. Due to the lockdown implemented in the middle of May of 2021, only a half influence of the disease effect was observed. Thus, we selected air pollutants and climatic factors from Taipei from June to July 2021 and compared them with the data from 2019.

The COVID-19 outbreak started in the Wanhua District in May 2021 in Taiwan, and the government implemented a lockdown policy in Taiwan beginning on 15 May 2021, which was in effect until 26 July 2021. We compared the rates of pediatric asthma ERVs between the pre-COVID-19 period (2019) and the COVID-19 pandemic (2020–2021). Air pollution and climatic factors were collected during the pre-COVID period, the COVID-19 pandemic, and COVID-19 pandemic with the lockdown period (second and third seasons of 2021). This study was approved by the Institutional Review Board of Tri-Service General Hospital of TSGHIRB-C202305039.

The Chi-square test was used to compare the rates of pediatric asthma ERVs during the COVID-19 and lockdown periods. Student’s *t*-test was used to compare air pollution and climatic factors before and after COVID-19, or during the lockdown period. The relations between pediatric asthma ERVs and the COVID-19, or the lockdown period, were determined using logistic regression analysis and reported as odds ratios (ORs). A Poisson analysis was performed to determine the relative risk (RR) of air pollution and climatic factors on pediatric asthma ERVs. The mean rates of pediatric asthma ERVs, air pollutant factors, and climatic factors for each season were plotted. Statistical significance was set at *p* < 0.05. All statistical analyses were performed using SPSS version 21.

## 3. Results

**Figure 1:**
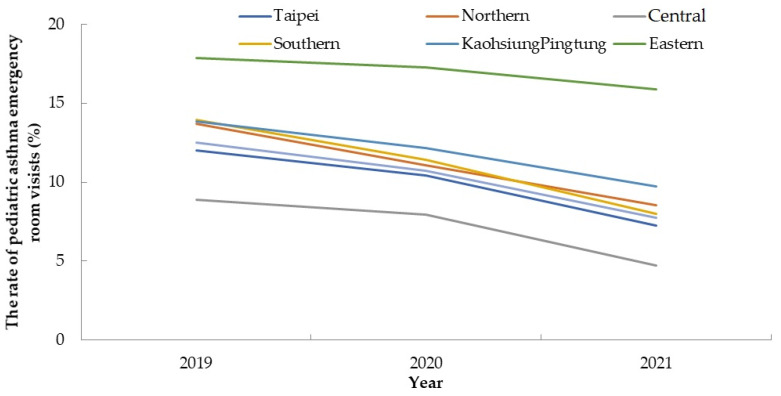
The rate of pediatric asthma emergency room visits in different areas.

The annual rate of pediatric asthma ERVs has decreased in the Taipei and Northern, Central, Southern, and Kaohsiung–Pingtung areas of Taiwan. However, there was no significant change in the rate of pediatric asthma ERVs in Eastern Taiwan during the COVID-19 pandemic. The ERV rates of pediatric asthma in different areas are shown in Figure 1. Changes in pediatric asthma ERVs rates are summarized in Table 1.

**Figure 2:**
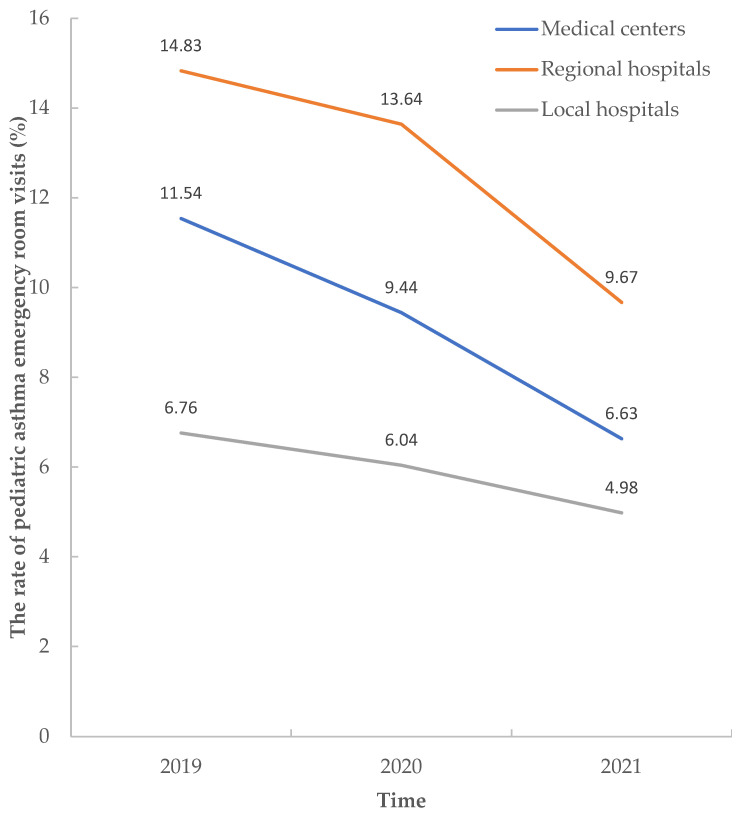
The rate of pediatric asthma emergency room visits in different hospital levels.

There was a significant reduction in pediatric asthma ERVs in 2020 and 2021 in medical centers, regional hospitals, and local hospitals compared to the rates in 2019 (pre-COVID-19). The rate of pediatric asthma ERVs ranged from 11.54% to 8.24% in medical centers (OR = 0.688; (95% CI: 0.644–0.735, *p* < 0.001). The rate of pediatric asthma ERVs ranged from 14.83% to 9.84% in regional hospitals (OR, 0.627; 95% CI: 0.589–0.667, *p* < 0.001). The rate of pediatric asthma ERVs in local hospitals ranged from 6.76% to 5.58% (OR, 0.815; (95% CI: 0.724–0.917, *p* < 0.001). The ERV rates of pediatric asthma decreased at different hospital levels (Figure 2).

With respect to air pollution in Taipei, the mean PM_2.5_ was 12.78 ± 2.8 µg/m^3^, the mean SO_2_ was 1.77 ± 0.33 ppb, the mean ozone was 26.45 ± 3.69 ppb, the mean NO_2_ was 18.04 ± 3.12 ppb, the mean air temperature was 24.14 ± 4.24℃, and the mean relative humidity was 75.28 ± 2.57%. The mean air pollutants and climatic factor levels between were not significantly different between the pre-COVID-19 and COVID-19 periods in Taipei.

The greatest air pollutant reduction was particulate matter ≤ 10 µm (PM_10_), which was 15.95%, followed by around 10% in SO_2_, CO, and NO_2_. The increase in O_3_ concentration was 3.36% (Table 2). Values with a higher Poisson’s correlation (*p* < 0.05) were excluded (Table 3). The adjusted RR of O_3_ was 1.094 (95% CI: 1.095–1.12, *p* < 0.001) per a 1 ppb increase in the COVID-19 era (Table 4). The adjusted RR of PM_2.5_ was 1.137 (95% CI: 1.042–1.239, *p* = 0.001) per a 1 µg/m^3^ increase in the pre-COVID-19 period.

## 4. Discussion

### 4.1. The Main Finding of This Study

The mean rate of pediatric asthma ERVs has decreased in the past 3 years and the decrease was significant in all regions except for the eastern area. In addition, the rates of pediatric asthma ERVs at medical centers, regional, and local hospitals have decreased significantly. Depending on the period, the mean seasonal rate of pediatric asthma ERVs was the lowest during the COVID-19 lockdown period, followed by the COVID-19 without lockdown period, compared to the pre-COVID-19 period in Taipei. Air pollutants PM_2.5,_ PM_10_, NO_2_, and CO decreased significantly during the COVID-19 lockdown period. Ozone was positively associated with pediatric asthma ERVs during the COVID-19 pandemic.

**Figure 3:**
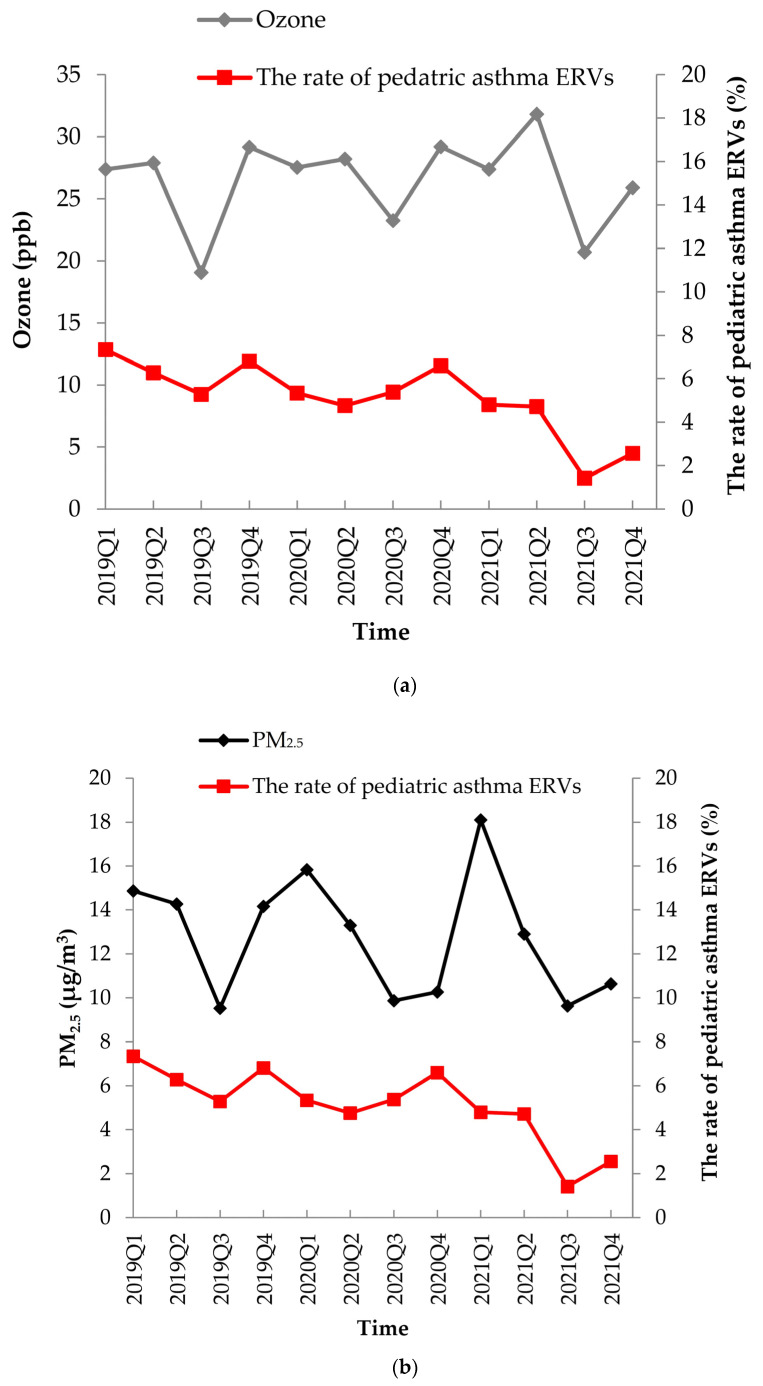

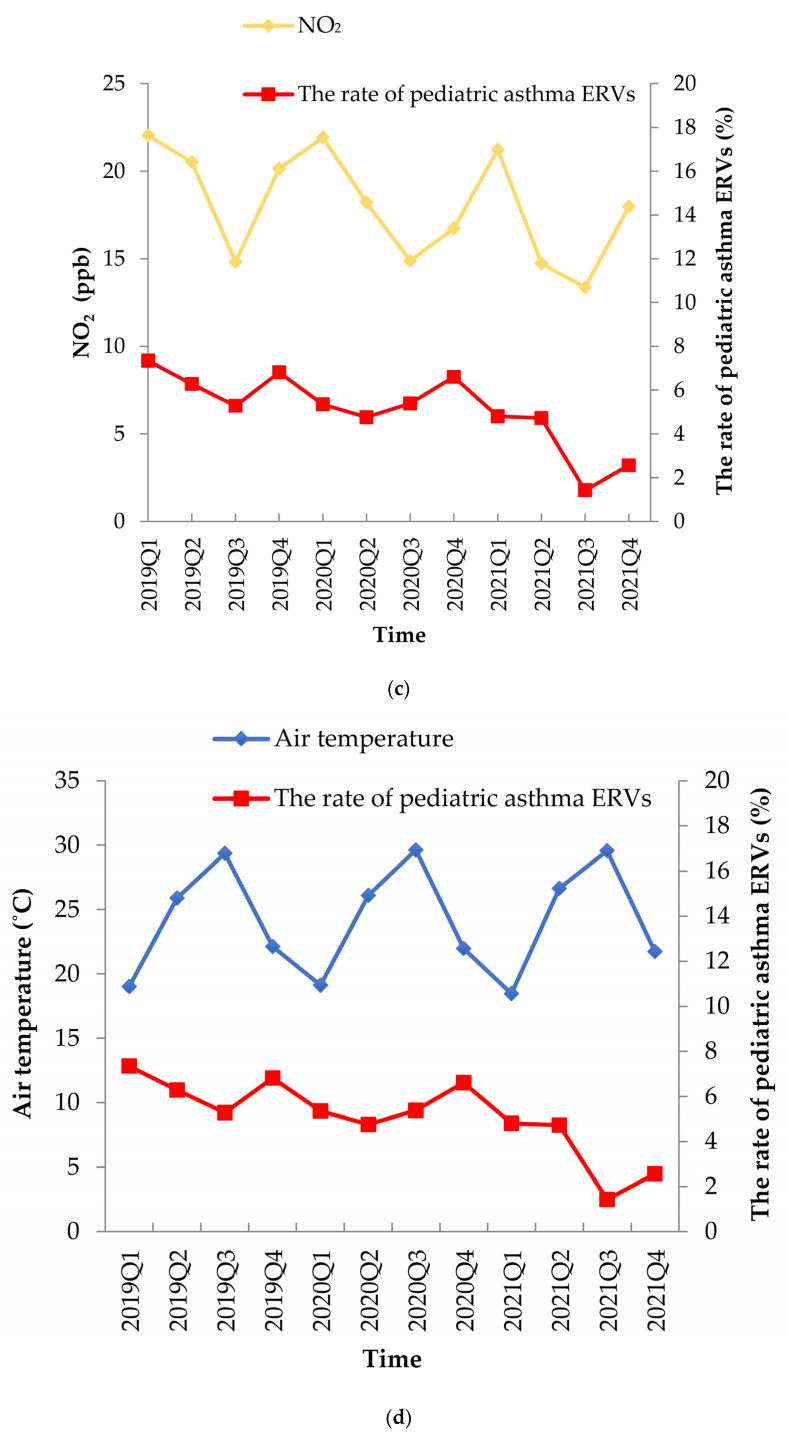
The air pollution (**a**) ozone, (**b**) PM_2.5_, (**c**) nitrogen dioxide, (**d**) air temperature, and the rate of pediatric asthma ERVs change from 2019 to 2021.

Pediatric asthma exacerbations decreased during the COVID-19 period in Northern Italy compared with the historical data [35]. In Canada, during the COVID-19 period, there were reductions in hospitalization (RR = 0.21), ERVs (RR = 0.35), and outpatient visits (RR = 0.61) in children and young adults with asthma [36]. The results of our study are similar and likely reflect the strict hygiene and social distancing measures adopted in 2020 during the COVID-19 pandemic. In addition, pediatric healthcare searches for serious outbreaks have decreased during the coronavirus pandemic. The decreasing trend in pediatric asthma ERVs was greater during the lockdown period (Figure 3). There were fewer ERVs which may indicate that the number of asthma attacks could be reduced or that asthma patients did not want to visit the emergency department with the potential risk of exposure to COVID-19. This was likely the result of a combination of related restriction measures. PM_2.5_, PM_10_, NO_2_, and CO levels decreased during the lockdown period, supporting the influence of restrictive measures. Several studies have shown that social distancing and increased hygiene measures reduce the diffusion of respiratory pathogens and decrease the incidence of respiratory tract infections. Additionally, adherence to asthma treatment increased during the lockdown period [40,41].

### 4.2. The Change in Air Pollution

The results show a clear decline in NO_2_ short-term levels and the annual average throughout Europe [42]. The rate of pediatric asthma ERVs was reduced, with the most in Southern Taiwan (OR = 0.68), and this reduction was related to improvements in air pollutant levels. Between 2019 and 2021 in Tainan, the air quality index (summed up by O_3_, PM_2.5_, PM_10_, CO, SO_2_, and NO_2_) [38] was decreased from 73 to 66, the PM_2.5_ decreased from 22.2 to 19.9 µg/m^3^, NO_2_ decreased from 11.18 to 10.29 ppb, and O_3_ decreased from 31.49 to 29.89 ppb [38]. There were no significant changes in the pediatric asthma ERVs in Eastern Taiwan. A potential reason for this finding is that there are fewer clinics in Eastern Taiwan, resulting in fewer patients being sent to the emergency department and lower air pollutant levels.

Between 2019 and 2021 in Hualien, the PM_2.5_ decreased from 8.5 to 7.8 µg/m^3^, NO_2_ decreased from 6.08 to 4.95 ppb, and O_3_ decreased from 29.49 to 27.07 ppb. The decrease in PM_2.5_ is likely due to fewer industrial factories, and the decrease in NO_2_ may be due to less residents and less transportation. We compared air pollutant levels in Taipei between the pre-COVID-19 and COVID-19 eras and found no significant decreases, but there were declining trends in PM_2.5_, PM_10_, and NO_2_ (Table 2). There was a significant decrease in air pollutant levels in Taipei during the lockdown period (Table 5). There was no significant reduction in pediatric asthma ERVs to local hospitals in 2020 compared to 2019, which is likely because, during the initial COVID-19 outbreak, patients were admitted to higher-level hospitals.

### 4.3. The Effect of Air Pollution in COVID-19 Period

There were 5275 COVID-19 patients in Taipei City and 7196 COVID-19 patients in New Taipei City during 2021. The majority of COVID-19 cases were in the Taipei metropolitan area, accounting for 76.5% (12,471/16,302) of cases in Taiwan [43]. We selected the Taipei metropolitan area to analyze the risk of pediatric asthma ERVs due to the severe COVID-19 pandemic and a similar rate of pediatric asthma ERVs in Taiwan [37]. Studies have reported that PM_2.5_, PM_10_, NO, NO_2_, and O_3_ reduce airway defense and induce inflammation and oxidative stress in the airways, which can easily trigger asthma exacerbation [13,14,15,16,17,22].

The cumulative risk of O_3_ on asthma exacerbation increased by 18.9% for 2 weeks [44]. Our results showed that ozone increased the risk of pediatric asthma ERVs, and there was a 9.4% increased risk of pediatric asthma ERVs for every 1 ppb increase in ozone. Our results showed a mildly increasing trend in the mean ozone level, as compared with the decreasing trends of the other air pollutants (Table 2). Possible reasons for the increase in ozone are the empowered photochemical reactions of increased global temperature [45], the development of the semiconductor industry [26], and the use of negative ion air purifiers and ozone machines for reducing pathogens during the COVID-19 pandemic in urban regions. The inverse effect showed the ozone levels and the number of ERVs exhibited a negative correlation in Korea [46]. A study found that PM_2.5_ and PM_10_ may have played important roles in severe pediatric respiratory events requiring ERVs in Kaohsiung during the COVID-19 pandemic [45]. Our results show that ozone increased the risk of pediatric asthma ERVs in Taipei, Taiwan. Since Kaohsiung City is an industrial production area in Taiwan, there are higher levels of air pollutants (2020; mean PM_2.5_ 20.1 µg/m^3^, PM_10_ 39.8 µg/m^3^, NO_2_ 12.55 ppb, and O_3_ 30.88 ppb), as compared to Taipei city that is an urban area where air pollution is primarily due to cars and residents (2020; mean PM_2.5_ 12.6 µg/m^3^, PM_10_ 23.6 µg/m^3^, NO_2_ 15.69 ppb, and O_3_ 29.98 ppb) [38]. The lower rate of childhood asthma exacerbations in a previous study paralleled the reduced TRAP levels observed during the pandemic. Possible reasons of reduction in O_3_, NO_2_, and PM_10_ during lockdowns have been widely studied in many locations worldwide. The reasons for this reduction are complex and include meteorological conditions. Although most previous studies have confirmed the decrease in NO_2_ during lockdowns, the decrease in PM_10_ and PM_2.5_ is less obvious and is often related to wind conditions. The past study found the lockdown response to COVID-19 has resulted in an unprecedented reduction in global economic activity and associated air pollutant levels, especially land transportation decline [32]. Fossil-fuel combustion was positively correlated with vehicular traffic in an urban context, where it showed decreased concentrations during the lockdown period [35]. Our study found a significant reduction in PM, NO_2_, and CO levels during the lockdown period, which potentially reduced the pediatric asthma ERVs.

The unscheduled asthma visits were reduced during COVID-19. It was potentially associated with reduced viral upper respiratory tract infections [47]; higher relative humidity increases pollen levels, increasing pediatric asthma exacerbations [48]. A relative humidity level of >60% in Taiwan resulted in more allergic diseases. Cumulative ambient exposure over 0–3 days to PM_2.5_ (odds ratio (OR) 1.01, per 6.2 µg/m^3^) and NO_2_ (OR 1.021 per 7.7 ppb) concentrations were associated with ERVs for COVID-19 [49]. Atmospheric PM_2.5_ concentrations are significantly associated with a greater risk of asthma exacerbation [13,14,15,16,17,50]. The short-term lag effect of PM_2.5_ was a 1.2-fold RR with a 2-day lag in pediatric asthma ED visits [20]. Our study found similar findings in the pre-COVID-19 period; however, PM concentration was not associated with pediatric asthma ERVs, since there was a decrease in the lockdown period due to the COVID-19 epidemic in Taipei. This can be explained by the complex formation chemistry in urban areas, mainly non-traffic sources combined with natural, anthropogenic, primary and secondary sources. In addition, the mean monthly PM_2.5_ decreased to below 10 µg/m^3^ during the lockdown period, reducing the number of pediatric ERVs for asthma. The potential reason for this was a reduction in winter wind pollution from mainland China during the COVID-19 pandemic because of factory closures and restrictive measures for citizens [45].

In this national retrospective cross-sectional study, we found that pediatric asthma ERVs were affected by the COVID-19 pandemic during 2020–2021, a major environmental trigger of ozone compared with PM_2.5_ before the COVID-19 period in urban areas. Restrictive measures implemented during the lockdown period significantly reduced air pollution, which potentially positively affected the incidence of pediatric asthma ERVs. The restrictive measures of public health interventions for COVID-19 reduced influenza virus infection [51], and lockdown measures such as school closures, stay-at-home orders, working at home, and protective measures (including social distancing, wearing masks, and increasing hand hygiene) have dictated changes in lifestyle, behavioral, and social habits, which are reflected in the reduced transmission of respiratory pathogens and reductions in anthropogenic atmospheric emissions [35]. Our study highlights the potential role of reduced exposure to air pollution with a slightly anomalous pattern of pediatric asthma ERVs in 2020–2021.

### 4.4. The Preventive Policy in Post-COVID-19 Era

The air pollutant at risk of pediatric asthma ERVs in Taiwan during the COVID-19 era was ozone. Carbon-containing masks reduce the effects of ozone on the respiratory tract. Residents were encouraged to use public transportation, drive electric cars or motorcycles, and ride bicycles to reduce TRAP. Other measures for reducing asthma attacks were promoted, such as using dehumidifiers to reduce the relative humidity to <50%, avoiding cold food, using non-negative ion air purifiers, avoiding smoking and second-hand smoke, regularly cleaning houses to reduce mite growth, regular outpatient follow ups, and adherence to controller medications.

## 5. Conclusions

This study demonstrates a reduction in pediatric asthma ERVs during the COVID-19 pandemic in Taiwan. Although decreased outside activity resulted in decreased air pollutants in the lockdown period, air pollutant levels did not change during 2020–2021. Ozone significantly increased the risk of pediatric asthma ERVs, without significant ozone elevation in the COVID-19 era. This research provides important information regarding PM_2.5_ reduction to a level that can reduce pediatric asthma ERVs, and ozone is still associated with health risks in childhood. An aggressive policy is needed to control the amount of air pollution and reduce ozone to decrease pediatric asthma ERVs, maintain national health, and protect the living environment to improve quality of life.

## 6. Limitations

This study has some limitations. First, the dataset did not contain the sex and age of the children. Children younger than six years are more sensitive to air pollution than those older than six years [51]. Second, detailed daily pediatric asthma treatments were not examined in this study, and air pollutants may have influenced the short-term frequency of asthma attacks. As seasonal pediatric asthma ERVs are supported by the government, we used seasonal data to survey these relationships during the COVID-19 period. Third, influenza infection decreased in a previous study [52]; however, a direct relationship between influenza infection and ERVs in pediatric asthma is lacking. The analysis should be adjusted for influenza if detailed data are available. The correlation between these two conditions should be investigated in the future. Fourth, the rate of smoking and second-hand smoke exposure were marked by juniors (2.2% cigarettes and 3.9% electronic cigarettes) and seniors (7.2% cigarettes and 8.8% electronic cigarettes) in high school by 2021 in Taiwan [53], which may increase pediatric asthma attacks and ERVs [17]. Registered studies are required to investigate the effects of smoking. Fifth, we used air pollution to assess the risk of pediatric asthma ERVs in Taipei; different areas may have different levels of pollution.

## Figures and Tables

**Table 1:** The rate of pediatric asthma emergency room visits during pre-COVID-19 period and COVID-19 period in Taiwan, the rate decline associated with COVID-19 period compared with pre-COVID-19 period using logistic regression.

Area	Pre-COVID-19	COVID-19	Odds Ratio (95% C.I.)	*p*
Overall	12.52%	9.47%	0.7558 (0.7254–0.7875)	<0.001 *
Taipei	12.02%	9.09%	0.7322 (0.6841–0.7837)	<0.001 *
Northern	13.71%	10.05%	0.703 (0.6438–0.7677)	<0.001 *
Middle	8.90%	6.48%	0.7095 (0.6310–0.7978)	<0.001 *
Southern	13.95%	9.90%	0.6776 (0.5966–0.7696)	<0.001 *
Kaohsiung–Pingtung	13.87%	11.14%	0.7788 (0.6967–0.8706)	<0.001 *
Eastern	17.87%	16.71%	0.9221 (0.7554–1.1256)	0.4253

* *p* < 0.05. C.I.: confidence interval.

**Table 2:** The mean air pollutants and climate factors between pre-COVID-19 and COVID-19 period in Taipei and the rate of seasonal pediatric asthma ERVs.

	Mean (2019–2021)	Pre-COVID-19 (2019)	COVID-19 (2020–2021)	Percentage of Reduction	*p*
SO_2_ (ppb)	1.77 ± 0.33	1.91 ± 0.19	1.7 ± 0.37	10.99%	0.221
CO (ppm)	0.43 ± 0.09	0.46 ± 0.82	0.41 ± 0.08	10.87%	0.323
O_3_ (ppb)	26.45 ± 3.69	25.87 ± 4.6	26.74 ± 3.47	−3.36%	0.753
NO_2_ (ppb)	18.04 ± 3.12	19.38 ± 3.16	17.38 ± 3.08	10.32%	0.337
PM_10_ (µg/m^3^)	25.32 ± 5.13	28.33 ± 6.1	23.81 ± 4.2	15.95%	0.246
PM_2.5_ (µg/m^3^)	12.78 ± 2.8	13.21 ± 2.47	12.57 ± 3.09	4.84%	0.708
Air temperature (°C)	24.14 ± 4.24	24.11 ± 4.49	24.15 ± 4.43	−0.17%	0.987
Relative humidity (%)	75.28 ± 2.57	75.67 ± 0.86	75.08 ± 3.16	0.78%	0.638
The mean rate of season pediatric asthma ERVs (%)	5.11 ± 1.71	6.43 ± 0.88	4.45 ± 1.66	30.79%	0.023 *

* *p* < 0.05.

**Table 3:** The correlation between air pollutants and climate factors in Taipei (COVID-19 period).

	SO_2_	CO	O_3_	NO_2_	PM_10_	PM_2.5_	Air Temperature	Relative Humidity
SO_2_	1	0.380	0.469	0.136	0.505	0.404	−0.058	−0.112
CO	0.38	1	0.306	0.932 **	0.913 **	0.878 **	−0.725 *	−0.012
O_3_	0.469	0.306	1	0.331	0.515	0.427	−0.44	0.394
NO_2_	0.136	0.932 *	0.331	1	0.831 *	0.822 *	−0.891 **	0.226
PM_10_	0.505	0.913	0.515	0.831 *	1	0.97 ***	−0.65	−0.002
PM_2.5_	0.404	0.878 **	0.427	0.822 *	0.97 ***	1	−0.695	0.106
Air temperature	−0.058	−0.725	−0.44	−0.891 **	−0.65	−0.695	1	−0.616
Relative humidity	−0.112	−0.012	0.394	0.226	−0.002	0.106	−0.616	1

* *p* < 0.05; ** *p* < 0.01; *** *p* < 0.001. SO_2_: sulfur dioxide; CO: carbon monoxide; O_3_: ozone; NO_2_: nitrogen dioxide; PM_10_: particulate matter ≤ 10 µm; PM_2.5_: particulate matter ≤ 2.5 µm.

**Table 4:** The relative ratio of pediatric asthma emergency room visits about air pollutants and climate factors in Taipei during COVID-19 period.

	Crude Relative Risk	*p*	Adjusted Relative Risk	*p*
PM_2.5_	1.012 (95% C.I.:0.997–1.027)	0.115	0.702 (95% C.I.:0.962–1.026)	0.702
Ozone	1.053 (95% C.I.:1.037–1.07)	<0.001 *	1.094 (95% C.I.:1.095–1.12)	<0.001 *
Air temperature	0.981 (95% C.I.:0.971–0.992)	0.001 *	1.018 (95% C.I.:0.985–1.052)	0.282
Relative humidity	1.017 (95% C.I.:1.003–1.031)	0.018 *	0.991 (95% C.I.:0.962–1.02)	0.536
COVID-19 without lock down	Reference		Reference	
COVID-19 with lock down	0.674 (95% C.I.:0.593–0.766)	<0.001 *	0.535 (95% C.I.:0.45–0.637)	<0.001 *
CO	3.094 (95% C.I.:1.758–5.446)	<0.001 *		
NO_2_	1.023 (95% C.I.:1.008–1.039)	0.003 *		
PM_10_	1.019 (95% C.I.:1.008–1.031)	0.001 *		

* *p* < 0.05. PM_2.5_: particulate matter ≤ 2.5 µm; CO: carbon monoxide; NO_2_: nitrogen dioxide; PM_10_: particulate matter ≤ 10 µm.

**Table 5:** The mean air pollutants and climate factors between pre-COVID-19 period and lockdown period in Taipei.

	Pre-COVID-19 Period	Lockdown Period	*p*
SO_2_ (ppb)	1.91 ± 0.23	1.75 ± 0.57	0.751
CO (ppm)	0.46 ± 0.09	0.27 ± 0.01	<0.001 *
NO_2_ (ppb)	19.38 ± 3.26	11.22 ± 0.02	<0.001 *
O_3_ (ppb)	25.87 ± 5.9	25.11 ± 3.49	0.821
PM_10_ (µg/m^3^)	28.33 ± 6.76	17.65 ± 0.35	<0.001 *
PM_2.5_ (µg/m^3^)	13.21 ± 3.07	9.1 ± 0.57	0.001 *
Air temperature (°C)	24.11 ± 4.46	29.8 ± 0.71	0.002 *
Relative humidity (%)	75.67 ± 2.23	73 ± 1.41	0.156

* *p* < 0.05. SO_2_: sulfur dioxide; CO: carbon monoxide; NO_2_: nitrogen dioxide; O_3_: ozone; PM_10_: particulate matter ≤ 10 µm; PM_2.5_: particulate matter ≤ 2.5 µm.

## Data Availability

The datasets used in the current study are available from the correspondence.
